# Supplementary material for: Novel role of the synaptic scaffold protein Dlgap4 in ventricular surface integrity and neuronal migration during cortical development
Source: Nat Commun. 2022 May 18;13:2746. doi: 10.1038/s41467-022-30443-z (PMC9117333; doi:10.1038/s41467-022-30443-z)
Supplement: Supplementary file 1 — Supplementary Information [file 41467_2022_30443_MOESM1_ESM.pdf]

Supplementary information

1. Clinical phenotypes of patients

P616

P616 is a French family and the subject is the first child born from a non-consanguineous couple, after 32 GW + 5 days. The patient presents cerebral paralysis after prematurity and a global delay of psychomotor acquisitions without intellectual disability. Exhibiting spastic diplegia, walking (digitigrade) was acquired at 18 months. A speech delay was observed and oral language lead to orthophonic care around 4 years. There were no seizure episodes. Weight-for-height growth is normal; the cranial perimeter is normal. Ophthalmologically, the child presented a nystagmus of regressive evolution the first months and developed severe hyperopia requiring appropriate management given the visual impairment. The child presents mixed developmental disorders involving those of the dyspraxic type, visuo-spatial and visuo-constructive disorders. There were phonological level difficulties in word repetition tests and in phonological awareness. These difficulties have repercussions on the written language at the syntactic, expressive and receptive level. The KABCII psychometric scale performed at the age of 10, taking into account the difficulties of visual support in view of the child's visual deficit, reveals heterogeneous but non-deficient results (NEMI II: cognitive efficiency index average: 88 <EC <100 or 34th percentile); verbal reasoning is normal. The child attended ordinary schooling in primary school and was referred to an ULIS (*Unités Localisées pour l'Inclusion Scolaire*) specialized school at the entrance to secondary school (11-12 years).

As well as the predicted mutation causing an intragenic insertion in *DLGAP*4 resulting in a frameshift (described in the text), other gene variations were identified by WES but considered unlikely to be pathogenic (**Suppl.Table 1-2**). These were in *ABCB10*, *ARX, TTN,* and *LRRK2* genes. Variations were associated with nonpathogenic phenotypes (*ARX*, Marques et al., 2015),

present in introns (*ABCB10*), or inherited substitutions without predicted consequence (*TTN* and *LRRK2*).

The splicing variant in the *ABCB10* gene was not considered as a potential candidate to explain the P616 brain malformation because numerous essential splicing variants exist for this gene in the gnomAD database. *TTN* and *LRRK2* are known disease-causing genes. The *TTN* gene was previously implicated in dominant cardiopathy and recessive muscular dystrophy (#MIM613765, 608807, 603689, 611705 and 600334). Mutation in the *LRRK2* gene is one of the most common causes of inherited Parkinson disease (#MIM607060).

P477

P477 is a family with monozygotic twins born from a non-consanguineous French family. There are also 4 healthy siblings in the family. Their father and one of his siblings had a history of benign epilepsy during infancy, without intellectual disability and a Chiari malformation was observed by MRI for the father. The neonatal period was normal. Neonatal parameters were within the normal range in both twins, with birth weight respectively 2690g and 2865g, length 51 cm in both sibs, and head circumference at 33 cm and 35 cm. Developmental delay became apparent from the age of 18 months. Neurological examination was normal except for permanent strabismus in both cases. They started walking at the age of 22 months. They developed severe speech delay, inability to read and write properly but they were autonomous in everyday life skills. Brain MRI performed at the age of 6 years old showed parieto-occipital pachygyria. At the age of 10.5 years, growth parameters were normal (weight respectively 29.7 kg and 27.2 kg, height 139 cm and 137.5 cm, and head circumference at 52 cm and 52.5 cm). Social interaction and behavior was normal. Neurological examination was normal. They have never experienced epileptic seizures. EEG showed focal spikes, located at the vertex in one and in the left fronto-central region in the other twin.

The P477 family shows a variation in *DLGAP4* (described in the text and in **Suppl. Tables 1-3**), inherited from the father, which is located downstream of the GH1 domain within the C-terminal region (the GH1 domain comprises the amino acids between Glu804 and Trp907 of DLGAP4 protein) (**Fig. 1b-c**) (Rasmussen et al. 2017). A single nucleotide substitution was identified by WES and considered likely to be pathogenic. There was also an inherited *DYNC*1H1 variation predicted to have no consequence (A. Carter, personal communication, https://www2.mrc-lmb.cam.ac.uk/groups/cartera/main.html), a *de novo* variation in *MYO1*C, of unknown consequence, and a variation in *ZFX* inherited from the unaffected mother. *MYO1C* and *ZFX* variants were not considered as primary candidates to explain the P477 brain malformation since dominant *MYO1C* mutations are known to cause bilateral sensorineural hearing loss in humans (Adamek et al., 2011; Lin et al., 2011) not present in patients identified here; and the *Zfx* knockout and conditional targeted mice present abnormalities in the hematopoiesis system (not affected in patients) but not in the nervous system (Jackson Laboratory), making it a less likely candidate.

Using Alamut software, we predicted the consequences of the two variants found in P477 and P616 on the three *DLGAP4* isoforms described by Ensembl and NCBI reference databases. In all transcripts, the P616 variant results in a frameshift mutation and the P477 variant in a Ser-Ala missense mutation. This information is described in **Suppl. Table 3**.

2. Generation of *Dlgap4*$^{tm1b(KOMP)Wtsi}$ mice and survival rate

The mouse *Dlgap4* gene is located on chromosome 2 and spans a genomic region of 150.7 kilobases. Mouse mutants were generated using the Knockout-first allele method (Skarnes et al, 2011) on the C57BL/6N background. The strategy relies on the identification of an exon common to all transcript variants (exon 8), upstream of which a LacZ cassette was inserted. Exon 8 of the *Dlgap4* allele was flanked by *loxP* sequences bilaterally (**Suppl. Fig. 10a**). The

resulting *Dlgap4*$^{tm1a(KOMP)Wtsi}$ mice were then exposed to Cre recombinase to create a non-conditional *lacZ*-tagged null allele without exon 8 removing the promoter-driven *neo* cassette. This generated the *Dlgap4*$^{tm1b(KOMP)Wtsi}$ mice that were then phenotyped (**Suppl. Fig. 10a**). At weaning age, mouse survival was assessed from 55 successfully genotyped mice originating from several different litters and derived from a heterozygous-by-heterozygous breeding scheme. We obtained the expected number of wild type and heterozygous mice, but no homozygous, suggesting that the *tm1b* allele of *Dlgap4* is not compatible with life. To determine the window of death, we carried out a recessive lethality screen at mouse embryonic day 14.5 (E14.5) and found 6 homozygous embryos amongst 35 collected (17%). The reason underlying lethality is unknown and will require further studies; however, these preliminary data suggest that death mainly occurs late during development between E14.5 and weaning age. Male and female heterozygous mice were studied independently and developed properly. Mouse weight did not significantly differ from their local matched controls.

3. Supplementary methods for *Dlgap4* mutant mice

Mice on the C57BL/6N background were maintained by the Mouse Genetics Project (MGP) Select pipeline at the Wellcome Sanger Institute (UK) and were given a breeders chow diet (Mouse Breeder Diet 5021, 9% crude fat content, 21% kcal as fat, 0.276ppm cholesterol, Labdiet, London,UK) from weaning. After weaning, animals were housed three to four mice per cage with WT controls housed separately, in specific-pathogen-free environment in individually ventilated cages under 12/12 light/dark cycle with temperature-controlled conditions and free access to food and water with hardwood bedding. All animals were regularly monitored for health and welfare concerns and were additionally checked prior to and after procedures.

We use a statistical model validated for comparison of a small number of knockout mice. More specifically, in inbred mutant mice, we are able to detect neuroanatomical defects with an effect size of 10% or more with 80% detection power using 3 mice per assessed groups (calculated using Gpower) (Collins et al. 2019).

Neuroanatomical studies

Neuroanatomical studies were carried out using 3 heterozygous knock-out *Dlgap4*$^{+/-}$ and 3 littermate WT mice at 16 weeks old as previously described (Collins *et al.*, 2018). Mice were anaesthetized with Ketamine (100 mg/kg, intraperitoneally) and Xylazine (10 mg/kg, i.p.), blood collected via the retro-orbital route and death confirmed before the brains were dissected and fixed in 4% buffered formalin for 48 hours, then transferred to 70% ethanol. Samples were embedded in paraffin using an automated embedding machine (Sakura Tissue-Tek VIP) and cut at a thickness of 5µm with a microtome in order to obtain sagittal brain section at Lateral +0.60 mm. The sections were then stained with 0.1% Luxol Fast Blue (Solvent Blue 38; Sigma-Aldrich) and 0.1% Cresyl violet acetate (Sigma-Aldrich) and scanned using Nanozoomer 2.0HT, C9600 series at 20× resolution. 40 brain parameters, made of area and length measurements as well as cell level features, were taken blind to the genotype across the sagittal section. Data were analyzed using a linear mixed model to determine whether a brain region was associated with neuroanatomical defect or not.

Ethical considerations in animal use

The care and use of mice in the Wellcome Sanger Institute study was carried out in accordance with UK Home Office regulations, UK Animals (Scientific Procedures) Act of 1986 under UK Home Office licence (80/2076) that approved this work, which was reviewed regularly by the Wellcome Sanger Institute Animal Welfare and Ethical Review Body.

4. References

1. Adamek N., Geeves MA, Coluccio, LM. Myo1c mutations associated with hearing loss cause defects in the interaction with nucleotide and actin. Cell. Mol. Life Sci. 2011; 68: 139–150.

2. Collins SC, Mikhaleva A, Vrcelj K. et al. Large-scale neuroanatomical study uncovers 198 gene associations in mouse brain morphogenesis. Nat Commun. 2019; 10: 3465.

3. Collins SC, Wagner C, Gagliardi L, Kretz PF, Fischer MC, Kessler P, et al. A Method for Parasagittal Sectioning for Neuroanatomical Quantification of Brain Structures in the Adult Mouse. Curr Protoc Mouse Biol. 2018; 8: e48.

4. Lin T, Greenberg MJ, Moore JR, Ostap EM. A hearing loss-associated myo1c mutation (R156W) decreases the myosin duty ratio and force sensitivity. Biochemistry. 2011; 50: 1831–1838.

5. Marques I, Sá MJ, Soares G, Mota Mdo C, Pinheiro C, Aguiar L, et. al. Unraveling the pathogenesis of ARX polyalanine tract variants using a clinical and molecular interfacing approach. Mol Genet Genomic Med. 2015; 3:203-214.

6. Skarnes WC, Rosen B, West AP, Koutsourakis M, Bushell W, Iyer V, et al. A conditional knockout resource for the genome-wide study of mouse gene function. Nature. 2011; 474: 337-342.

7. Rasmussen AH, Rasmussen HB, Silahtaroglu A. The DLGAP family: neuronal expression, function and role in brain disorders. Mol Brain. 2017; 10: 43.

5. Supplementary Tables and Figures

Supplementary Table 1. Summary of whole exome sequencing variants found altered in P477 and P616 probands.

Family	Gene	Genomic position	Mode of transmission	Dbsnp	Reference	cDNA	Protein	Polyphen	Sift	Inherited from	pLi score[3]	Missense Z score[3]	Minor allele frequency[3]
P477	*MYO1C*	17_1371366_G_A	*De novo*	rs761228547	NM_001080779.1	c.2812C>T	p.Arg938Trp	Probably Damaging	Deleterious		0	-0,64	1.59e-5
	DLGAP4	20_35155357_T_G	Inherited	rs1371852290	NM_014902.4	c.2893T>G	p.Ser965Ala	Probably Damaging	Tolerated	Father	0.99	2,76	3.2e-5
	DLGAP1	18_3534398_T_C	Inherited	rs752804789	NM_001242766.1	c.1397A>G	p.Asp466Gly	Probably Damaging	–	Mother	1	3,2	1.99e-5
	DYNC1H1 [1]	14_102500377_G_T	Inherited		NM_001376.4	c.10478G>T	pSer3493Ile	Probably Damaging[1]	Deleterious [1]	Father	1	10,97	0
	ZFX	X_24197602_G_C	Inherited	rs138812925	NM_003410.3	c.361G>C	p.Asp121His	Probably Damaging	Deleterious	Mother	1	3,15	1.09e-5
P616	*ABCB10*	1_229693882_C_T	*De novo*	rs1663305011	NM_012089.2	c.517+1G>A	Predicted splice site?	–	–		0	1,35	0
	DLGAP4	20_35154373_35154374 insCAGCTGG	*De novo*		NM_014902.5	c.2714_2715 ins CAGCTGG	p.Asn905Gln_fs	–	–		0.99	2,76	0
	ARX [2]	X_25031657_25031671 del	*De novo*	rs750585274	NM_139058.2	c.441_455del	p.Ala151Ala155del	–	–		0,91	2,05	0
	TTN	2_179596269_G_A	AR[4] compound	rs72648943	NM_133378.4	c.13492C>T	p.Leu4498Phe	Probably Damaging	Benign	Mother	0	-1,1	3.09e-4
		2_179401935_C_T		rs72648278	NM_133378.4	c.92197G>A	p.Glu30733Lys	Probably Damaging	Benign	Mother			3.27e-4
		2_179494077_A_T		rs747654057	NM_133378.4	c.36671T>A	p.Ile12224Asn	Probably Damaging	Benign	Father			2.82e-5
		2_179397654_A_G		rs55945684	NM_133378.4	c.95984T>C	p.Val31995Ala	Probably Damaging	Benign	Mother			5.49e-4
	LRRK2	12_40634345_C_T	AR compound	rs112794616	NM_198578.3	c.632C>T	p.Ala211Val	Benign	Benign	Father	0	1,26	1.74e-4
		12_40687428_G_A		rs200795874	NM_198578.3	c.2771G>A	p.Arg924His	Benign	Benign	Mother			7.58e-5

1. Predicted by X-ray crystallography to not greatly impact protein function. Personal communication, A. Carter. https://www2.mrc-lmb.cam.ac.uk/groups/cartera
2. Marques et al. Unraveling the pathogenesis of ARX polyalanine tract variants using a clinical and molecular interfacing approach. Mol Genet Genomic Med. 2015. 3(3):203-14. *Non pathogenic variant.*
3. gnomAD v2.1 was used for variant filtering. The pLI score reflects the tolerance of a given gene to the loss of function on the basis of the number of protein truncating variants, that is, the frameshift, splice donor, splice acceptor, and stop-gain variants referenced for this gene in control databases weighted by the size of the gene and the sequencing. In research and clinical interpretation of Mendelian cases, pLI > 0.9 is widely used for prioritizing variants. Deviation from expectation is predicted with a Z score (Fu et al., 2013), which for synonymous variants is centred at zero, but is significantly shifted towards higher values (greater constraint) for both missense and protein-truncating variant (Wilcoxon P < 10−50 for both, Lek et al, 2016). A minor allele frequency of <0.1 is considered as rare. Fu, W. et al. Analysis of 6,515 exomes reveals the recent origin of most human protein-coding variants. Nature.2013. 493, 216–220; Lek, M., Karczewski, K., Minikel, E. et al. Analysis of protein-coding genetic variation in 60,706 humans. Nature.2016. 536, 285–291.
4. AR: autosomal recessive

Supplementary Table 2. Summary of whole exome sequencing information of the two family trios: P477 and P616.

**Family**	**P477**			**P616**		
Individuals	Father	Mother	Proband	Father	Mother	Proband
Clinical status	unaffected	unaffected	affected	unaffected	unaffected	affected
average depth for nucleotides in captured region	95,4	106	126,8	147,5	140	156,9
% of captured regions with coverage >15	96,7	93,1	98,5	98,8	98,6	99
% of captured regions with coverage >30	88,9	86,5	94,6	95,6	95,1	96,3
Total number of SNPs (essential splicing and coding regions) before filtering	14449	14517	14369	14621	15077	14873
Total number of INDELs (essential splicing and coding regions) before filtering	886	862	866	828	824	828
Total number of SNPs after filtering	249	253	270	203	231	217
Total number of INDELs after filtering	18	15	19	20	21	27
Total number of genes with *de novo* variants after filtering	-	-	1	-	-	3
Total number of genes with recessive variants after filtering	-	-	-	-	-	2

SNP, single nucleotide polymorphism

INDEL, insertion or deletion

Supplementary Table 3. Mutations detected in the different transcripts of *Dlgap4.*

Patient	Reference		Exon	Bp	Aa	cDNA	Protein	Sift	MT	Polyphen2
P616	NM_014902.5	ENST00000373913.7	12	5056	989	c.2714_2715insCAGCTGG	p.Asn905Gln_fs*100	/	/	/
	NM_183006.3	ENST00000340491.8	7	2392	453	c.1107_1108insCAGCTGG	p.Asn369Gln_fs*100	/	/	/
	NM_001042486.3	ENST00000475894	7	3110	285	c.603_604insCAGCTGG	p.Asn201Gln_fs*100	/	/	/
P477	NM_014902.5	ENST00000373913.7	13	5056	989	c.2893T>G	p.Ser965Ala	Tolerated	Disease causing	Probably damaging
	NM_183006.3	ENST00000340491.8	7	2392	453	c.1285T>G	p.Ser429Ala	Tolerated	Disease causing	Probably damaging
	NM_001042486.3	ENST00000475894	7	3110	285	c.781T>G	p.Ser261Ala	Tolerated	Disease causing	Probably damaging

Bp, base-pair ; Aa, amino acid ; MT, Mutation Taster

Supplementary Table 4. Nucleotide sequences of the primers used in the different approaches performed in this study.

**Primer name**	**Nucleotide sequence**
In situ hybridization	
Dlgap4F	GGAATTACCCCAGGCCCTGAA
Dlgap4R	GTTGGCCTTGAGGTGGTAGAG
RT-qPCR	
Dlgap4F	CTCCCGCAGCCGCATC
Dlgap4R	AGAGGGGAGGCACGGG
CypF	CCATCGTGTCATCAAGGACTT
CypR	TTGCCATCCAGCCAGGAGGTC
IUE Sh or Scrambled sequences	
ShRNA*Dlgap4*	TACCCTTCAGTATGGTTATTA
ShRNA*Control* [1]	TCTTAATACCTGCGATTTCAT

RT-qPCR, reverse transcribed quantitative PCR
Sh, short hairpin
[1] This is a scrambled sequence generated from the specific ShRNA*Dlgap4* sequence using Wizard (https://www.invivogen.com/sirnawizard/scrambled.php)

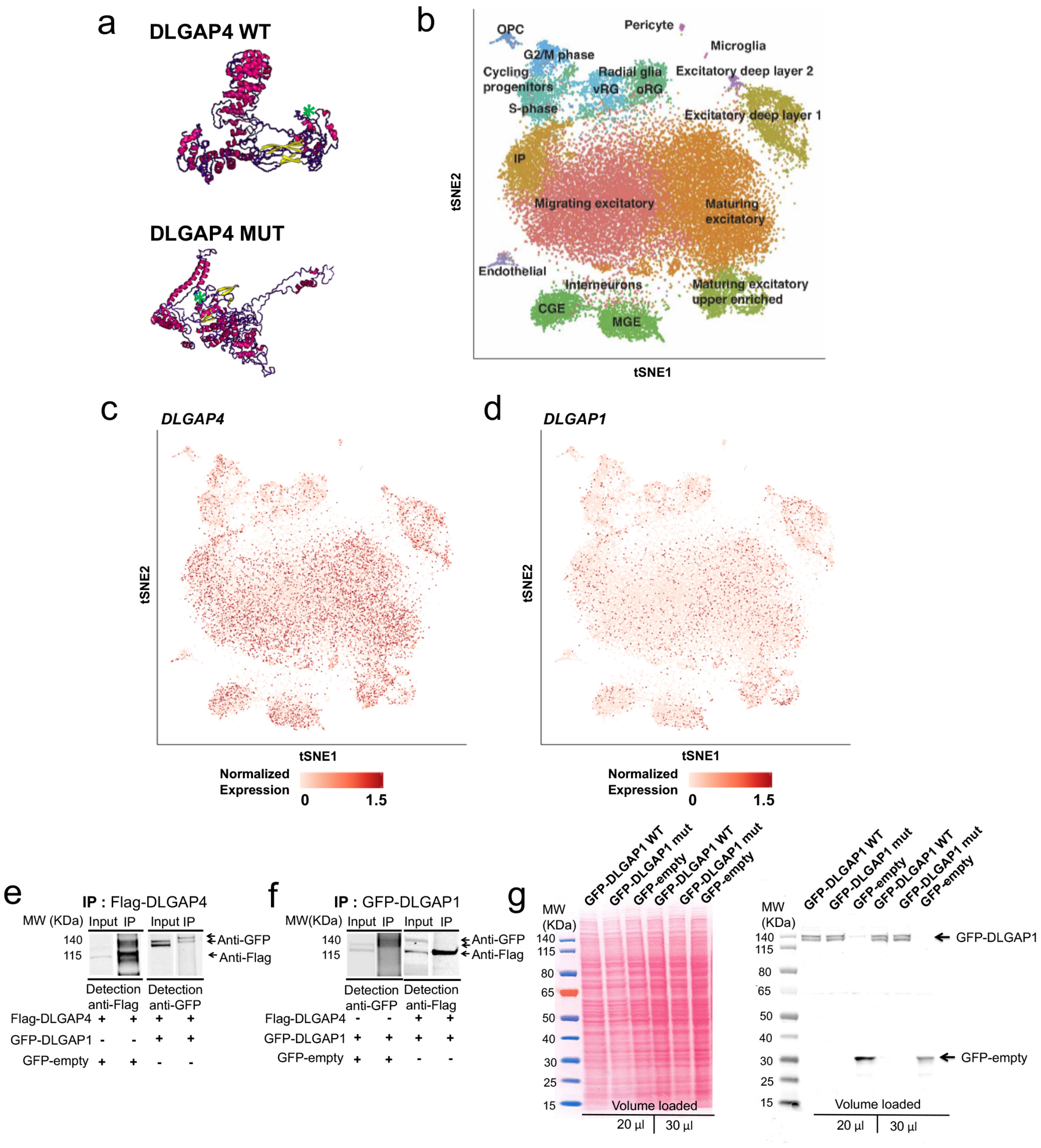

Supplementary Figure 1. Predicted models by I-TASSER. *DLGAP1* and *DLGAP4* expression in humans and Co-IPs. (a) I-TASSER 3D predicted structure of WT and mutant proteins focusing on the region of the mutation. Best predicted models are shown for DLGAP4 (WT and P616 mutation). I-TASSER cartoon predicted models are: WT DLGAP4: C-score -2.88, estimated RMSD 16.3±3.0Å and estimated TM-score 0.39±0.15; P616 mutation DLGAP4: C-score -1.28, estimated RMSD 12.1±4.4Å and estimated TM-score 0.56±0.15. **(b)**

The T-distributed Stochastic Neighbor Embedding (tSNE) tool shows the mRNA sequencing (scRNA-seq) expression of individual human embryonic cells. tSNE: each point on the left graph represents a cell and the relative closeness of cells to each other indicates underlying similarity between them. By unbiased clustering, transcriptionally distinct cell clusters show the main categories, e.g. astrocytes, oligodendrocyte precursor cells, microglia, radial glia, intermediate progenitor cells, excitatory cortical neurons, ventral MGE progenitors, inhibitory cortical interneurons, choroid plexus cells, mural cells, and endothelial cells. (**c-d**) The scatterplot for *DLGAP4* and *DLGAP1* was performed from the database generated by Polioudakis et al. (2019), after principal components analysis and tSNE algorithm application. The normalized expression level is represented through a white to red gradient. Source: http://solo.bmap.ucla.edu/shiny/webapp/#. (**e-f**) DLGAP4 and DLGAP1 are present in the same precipitates. Co-IPs were performed from Neuro2A cell extracts co-transfected with Flag-DLGAP4 and GFP-DLGAP1 and work in both directions (n=2 experiments per condition). (**g**) Total protein and immunoblot from Neuro2a cell extracts transfected with WT and P477 mutant DLGAP1 constructs show no signs of mutant protein degradation nor decreased levels at the specific band molecular weight compared to WT protein. The change from an acidic to a non-polar and the smallest in size amino acid, might destabilize certain protein-protein interactions. I-TASSER: Iterative Threading ASSEmbly Refinement; C-score: confidential score; RMSD: Root Mean Square Distance, average distance of all residue pairs in two structures; TM-score: scale for measuring the structural similarity between two structures.

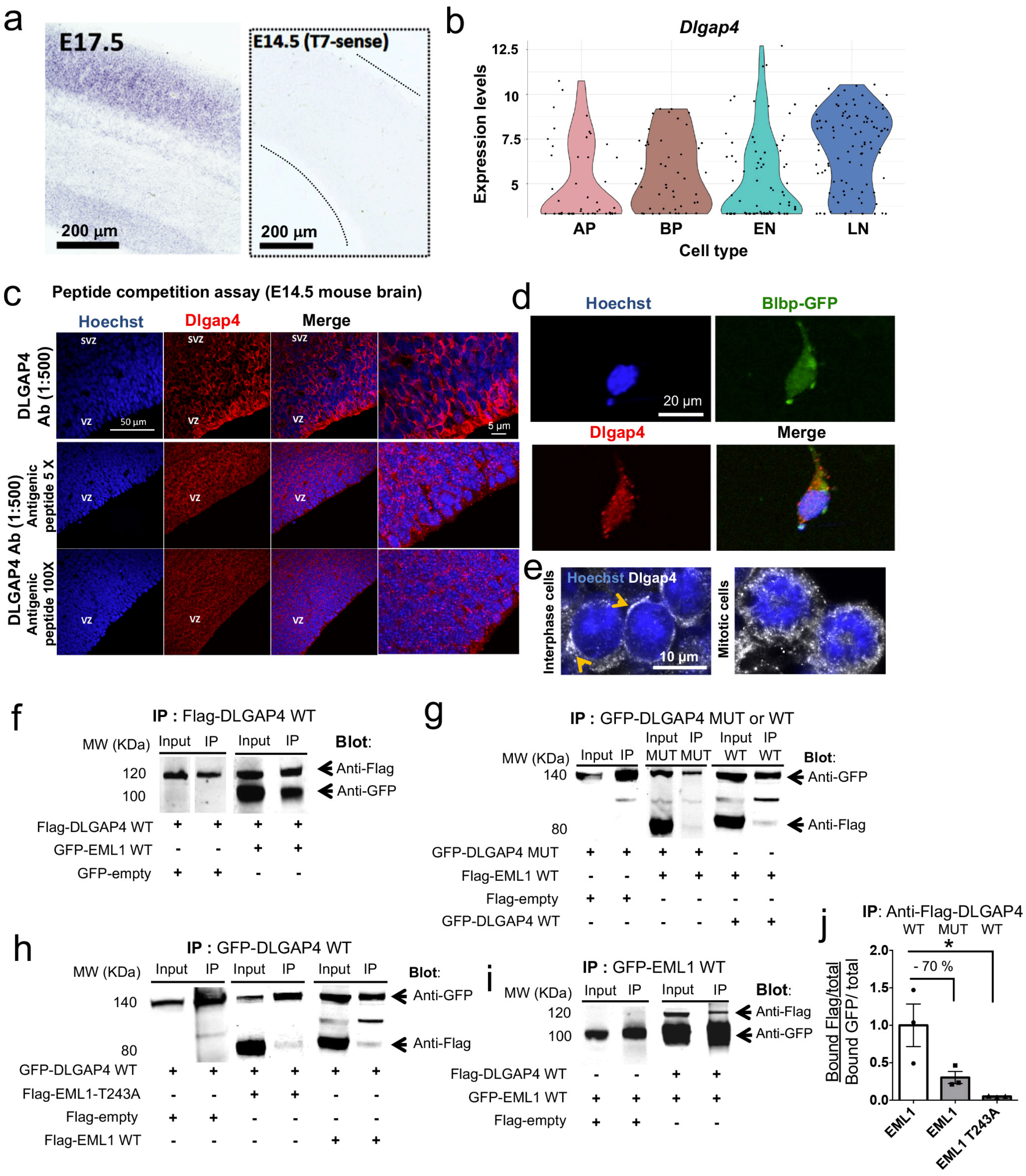

Supplementary Figure 2. Expression of *Dlgap*4/Dlgap4 during mouse cortical development. DLGAP4 co-IP with EML1 and patient mutations impair their binding. (**a**) *In situ* hybridization (ISH) at E17.5 shows expression of *Dlgap*4 throughout the cortical wall and strongest in the VZ and CP. The sense probe shows no labeling. (**b**) Single-cell transcriptomic analysis of the developing mouse neocortex (E14.5) atlas shows the expression pattern as a violin graph-plot of *Dlgap4* transcripts among cell types. AP: Apical progenitors;

BP: daughter basal progenitors; EN: Early neurons; LN: Late neurons. Source: http://genebrowser.unige.ch/science2016/. (**c**) Peptide competition assay shows the specificity of the antibody used in immunohistochemistry at E14.5. Two different concentrations of the commercial antigenic peptide were evaluated (5X and 100X), both showing a strong reduction in specific signal (compare with upper panel). (**d**) Endogenous Dlgap4 expression in primary cultures of cortical progenitors transfected with a Blbp-GFP construct. (**e**) Endogenous Dlgap4 expression in Neuro2A cells both in interphase (left) or mitotic (right) cells. Yellow arrows show regions of protein enrichment. (**f-i**) IP analyses were performed in Neuro2A cells, co-transfected with vectors expressing tagged EML1 and DLGAP4. Total extracts were either loaded directly on the gel (Input) or subjected to IP with anti-Flag or anti-GFP antibodies as indicated above each blot. Representative blots are shown for all conditions. (**f-i**) EML1 and DLGAP4 co-immunoprecipitate (co-IP). Several conditions were tested: Flag-DLGAP4 and GFP-EML1 co-IP using either anti-Flag (**f**) or anti-GFP (g-i) for the IP. WT or mutant (T243A) Flag EML1 co-IP was tested with either MUT (**g**) or WT GFP-DLGAP4 (**h-i**). (**j**) Quantification data represent the relativized individual values, means ± SEM (n=3 independent experiments per condition). One-way ANOVA: $F_{2,6} = 8.34$, $p = 0.019$; WT DLGAP4 vs MUT DLGAP4's EML1 IP: $p = 0.062$, WT DLGAP4 vs WT DLGAP4's mutant EML1 IP: $^{*}p < 0.05$, by *post hoc* Tukey's test.

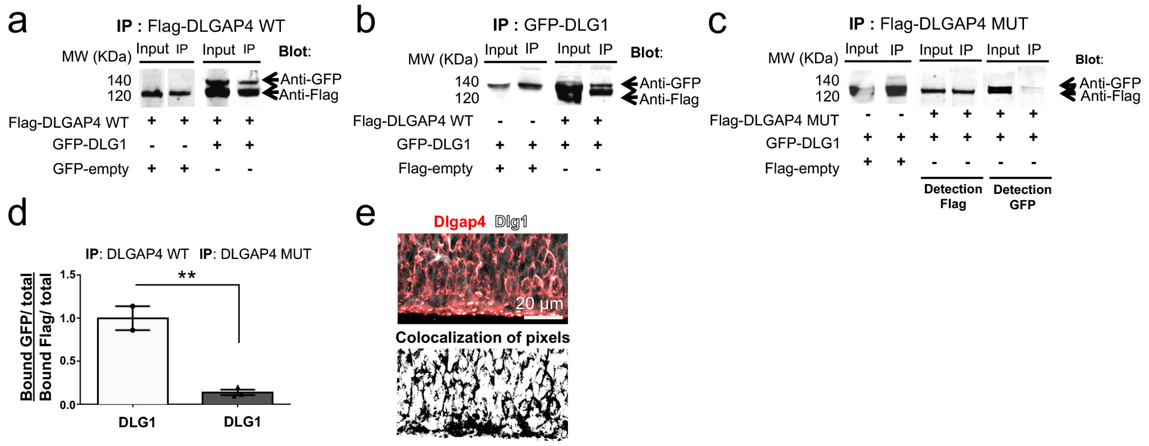

Supplementary Figure 3. Dlgap4 co-IP with DLG1. (a-c) Analyses show that Flag-DLGAP4 and GFP-DLG1 co-IP using either anti-Flag (**a, c**) or anti-GFP (**b**) for the IPs. (**d**) Representative blots show reduced levels of DLG1 in MUT DLGAP4 IPs. Quantification graph shows the relativized individual values, mean ± SEM. Two-sided unpaired t-test was performed (n=2 WT DLGAP4, n=3 MUT DLGAP4 independent experiments per condition, ** *p = 0.0044*). (**e**) Co-localization of Dlg1 and Dlgap4 in E14.5 mouse neocortex with Pearson's correlation coefficient (mean ± SEM) r= 0.62 ± 0.067 (n=3 WT brain embryos).

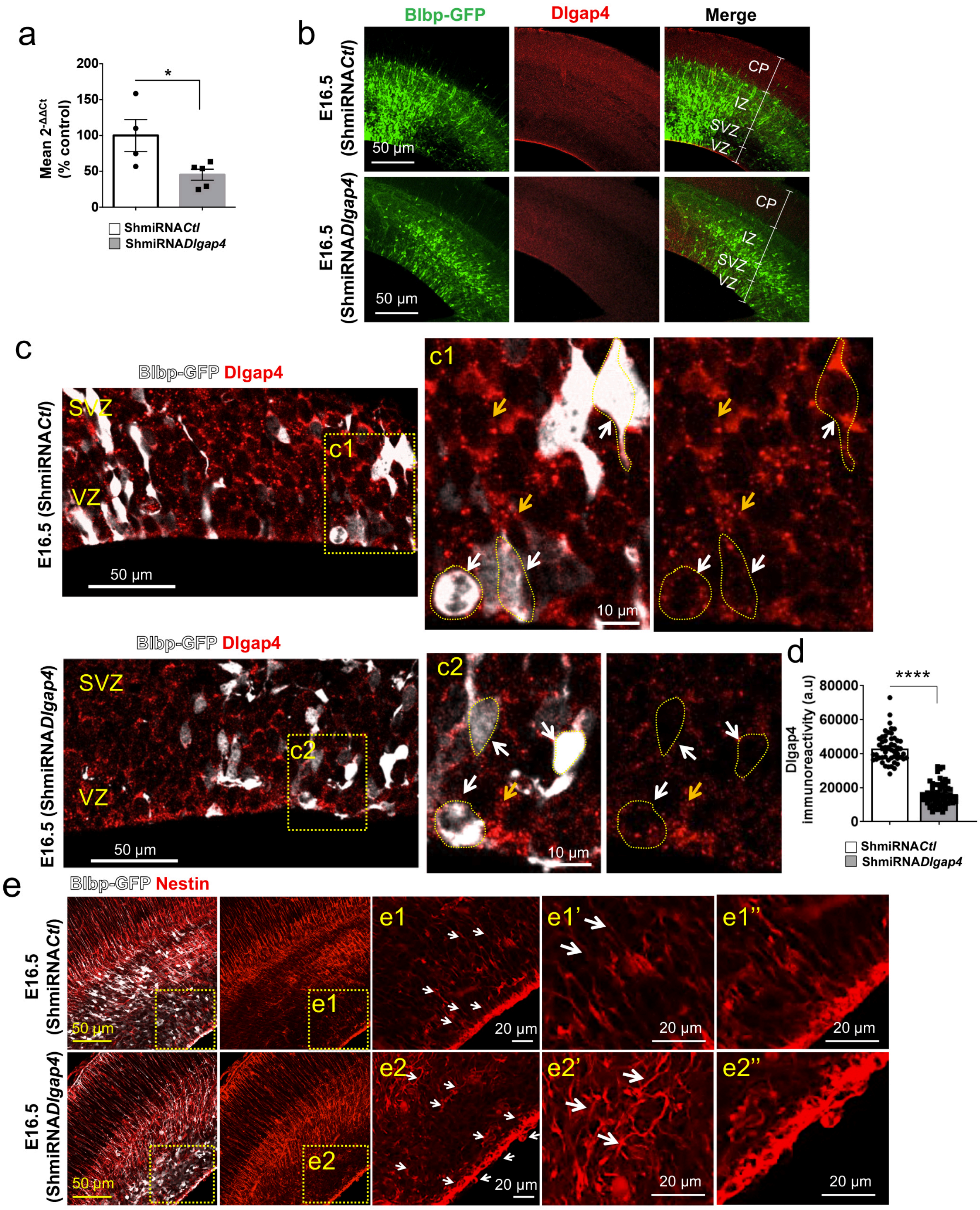

Supplementary Figure 4. **Validation of the ShmiRNA*Dlgap4* construct by RT-qPCR and immunofluorescence**. **RG morphology is affected.** (**a**) *Dlgap4* KD by specific ShmiRNA showed a significant reduction of mRNA expression performed by plasmid transfection in Neuro2A cells followed by RT-qPCR. Quantification data represents relativized individual

values, mean ± SEM (n=4 *Ctl, n=5 Dlgap4* KD independent cell cultures per condition). Two-sided unpaired t-test, * $p = 0.039$ was performed. (**b**) Low magnifications of representative images of E16.5 mouse neocortex co-electroporated at E14.5 with ShmiRNA*Ctl* or ShmiRNA*Dlgap4* and Blbp-GFP constructs. Immunostainings correspond to anti-Dlgap4 (red) and anti-GFP (green) detections. Different cortical regions are indicated. (**c**) Immunofluorescence images show the KD of *Dlgap4* by specific ShmiRNA. Compare yellow arrows (non-electroporated cells) with white arrows, showing Blbp-GFP (white) and ShmiRNA co-electroporated cells. (**d**) Quantification of data show a significant reduction of Dlgap4 immunoreactivity. Results are represented by the individual values, mean ± SEM (52 cells from n=2 ShmiRNA*Ctl* brains and 59 cells from n=3 ShmiRNA*Dlgap4* brains, two-sided unpaired t-test, **** $p < 0.0001$). (**e**) Analysis of progenitor morphology in IUE brains for control (Shmi*Ctl*, upper) and *Dlgap*4 KD (Shmi*Dlgap*4, lower) constructs reveal disorganized nestin$^+$ RG fibers (red) close to the ventricular surface (compare white arrows indicating nestin$^+$ RG fibers). Different higher magnification regions from ShmiRNA*Ctl* (**e1, e1' and e1''**) and ShmiRNA*Dlgap4* electroporated brains (**e2, e2' and e2''**) are indicated.

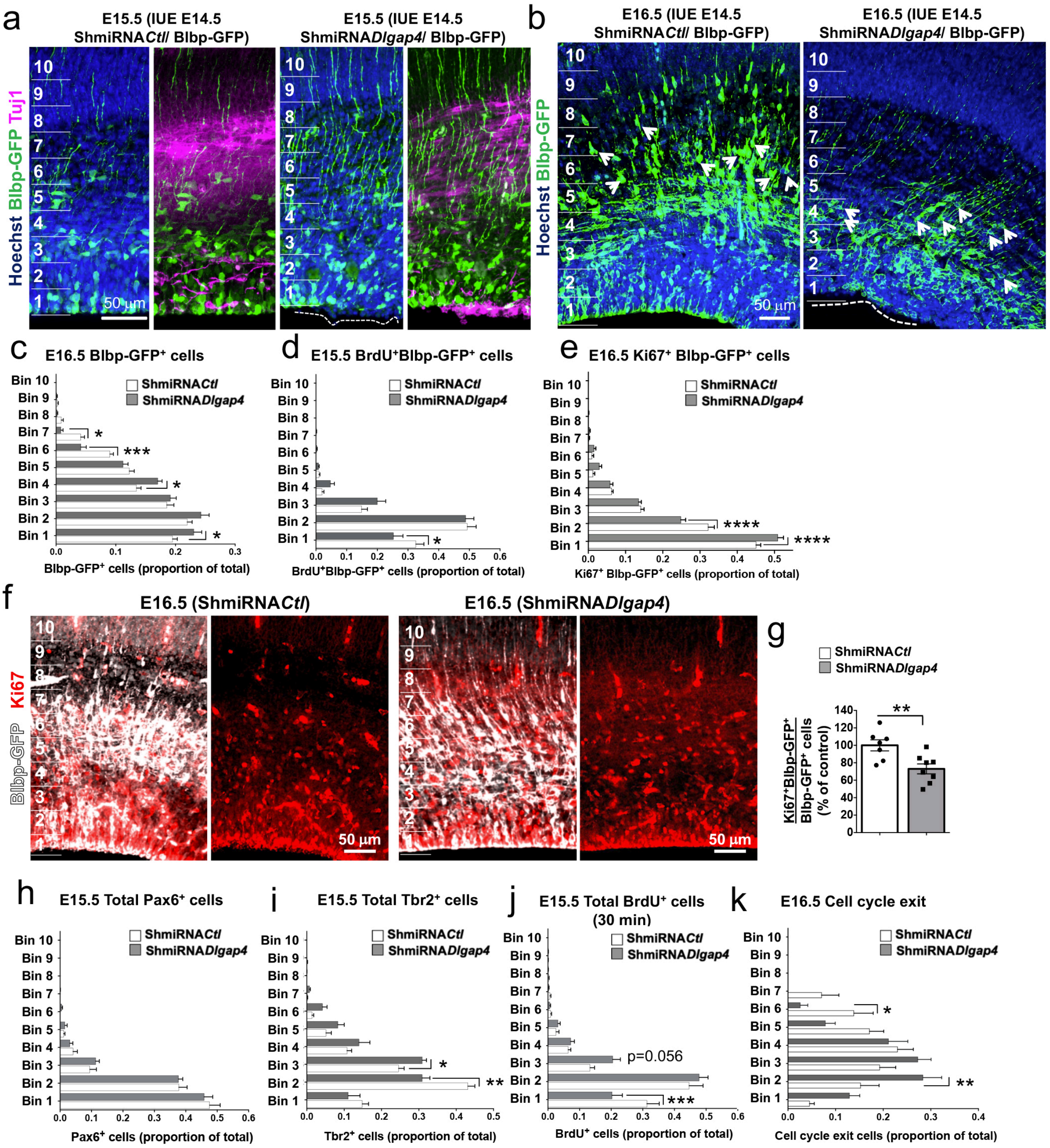

Supplementary Figure 5. ***Dlgap4*** **KD shows a strong ventricular surface phenotype with ectopic TuJ1$^+$ cells, abnormal cell distribution and proliferation defects.** (**a**) Representative images of control (Shmi*Ctl*, left) and *Dlgap*4 KD (Shmi*Dlgap*4, right) show ectopic TuJ1$^+$ (magenta) cells in mutant KD brains (E15.5). (**b**) A ventricular surface phenotype is also evident 48 h after IUE in the KD condition. Arrows: more cells in apical bins than migrating basally. (**c**) An abnormal distribution of Blbp-GFP$^+$ (green) progenitor cells shows delayed migration in *Dlgap4* KD (see Bin 4, 6 and 7). Results are represented by mean ± SEM (n=5

Shmi*Ctl*, n=6 Shmi*Dlgap4* embryos). Two-way ANOVA followed by Sidak's multiple comparisons test was performed. Interaction Bin x ShmiRNA condition, $F_{9, 90}$= 6.04, $p < 0.0001$; * $p < 0.05$, *** $p = 0.0003$. (**d-e**) Quantification of $BrdU^+$Blbp-GFP^+ and $Ki67^+$ Blbp-GFP^+ cells along the cortical wall. Two-way ANOVA followed by Sidak's multiple comparisons test was performed. For BrdU (n=12 Shmi*Ctl*, n=15 Shmi*Dlgap4* embryos): Interaction Bin x ShmiRNA, $F_{9, 250}$= 2.02, $p = 0.038$; * $p = 0.01$; for the proliferation marker Ki67 (n=7 Shmi*Ctl*, n=10 Shmi*Dlgap4* embryos): Interaction Bin x ShmiRNA, $F_{9, 150}$= 8.23, $p < 0.0001$; **** $p < 0.0001$. (**f-g**) Representative images and quantification data of $Ki67^+$ co-labeled with Blbp-GFP, 48h after IUE (n=7 Shmi*Ctl*, n=8 Shmi*Dlgap4* embryos), mean ± SEM (two-sided unpaired t-test, ** $p = 0.0073$). (**h-k**) Quantifications of total $Pax6^+$, $Tbr2^+$, $BrdU^+$ and $Ki67^-BrdU^+$Blbp-GFP^+/$BrdU^+$ Blbp-GFP^+ cell distribution. Analyses correspond to two-way ANOVA followed by Sidak's multiple comparisons test. For $Pax6^+$ (n=6 Shmi*Ctl*, n=7 Shmi*Dlgap4* embryos): neither the interaction (bin x ShmiRNA) or main effects (ShmiRNA) were significant, $F_{9, 120}$ = 0.25, $p = 0.99$; for Tbr2 (n=6 Shmi*Ctl*, n=5 Shmi*Dlgap4* embryos): Interaction Bin x ShmiRNA, $F_{9, 90}$= 6.66, $p < 0.0001$; * $p = 0.019$, **** $p < 0.0001$; for BrdU (n=7 Shmi*Ctl*, n=8 Shmi*Dlgap4* embryos): Interaction Bin x ShmiRNA, $F_{9, 120}$= 3.16, $p = 0019$; $p = 0.056$, *** $p = 0.0003$; for cell cycle exit ($Ki67^-BrdU^+$Blbp-GFP^+/$BrdU^+$Blbp-GFP^+) (n=7 Shmi*Ctl*, n=10 Shmi*Dlgap4* embryos): Interaction Bin x ShmiRNA $F_{9, 150}$= 4.97, $p < 0.0001$; * $p = 0.019$, ** $p = 0.0035$.

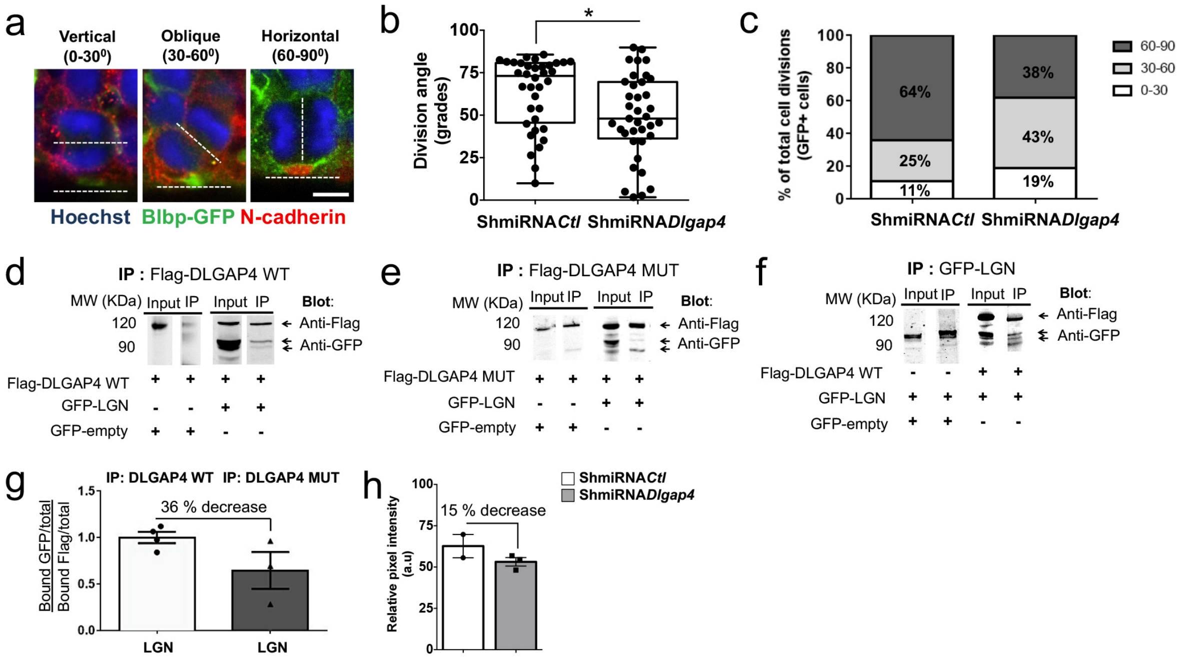

Supplementary Figure 6. Analysis of division angle of cortical progenitors in electroporated brains. (**a**) Representative Blbp-GFP^{+} anaphase cell images are shown at the ventricular surface, with vertical, oblique and horizontal divisions, as indicated with dotted lines. Representative images from E15.5 brains (IUE at E14.5). Scale bar: 5 µm. (**b**) Division angle median is lowered in the KD condition (n=2 embryos, 36 Blbp-GFP^{+} cells per condition). Data are shown as median (73.09 and 48.03) with box bounds at 25% (45.59 and 36.31) and 75% percentile (80.71 and 69.59), whiskers show maxima (85.68 and 89.83) and minima (9.91 and 1.70). Two-sided Mann Whitney test was performed, * $p = 0.012$. (**c**) More vertical and oblique divisions are observed in the KD condition at the expense of horizontal. (**d, e**) Co-IP analyses show that GFP-LGN is present in WT Flag-DLGAP4 (**d**) and mutant (**e**) precipitates. (**f**) Flag-DLGAP4 is also found in GFP-LGN precipitates. (**g**) Graph showing the amount of total GFP-LGN precipitated, comparing WT DLGAP4 (n = 3) and mutant (n = 4) independent experiments. Quantification graphs show the relativized individual values, mean ± SEM. Statistical analysis was performed using two-sided unpaired t-test with Welch's correction, *n.s.* $p = 0.21$. (**h**) The mean intensity of F-actin fluorescence was analyzed after *en face* imaging,

showing a possible tendency for reduction (*n.s, p=0.39*). No visible ventricular surface damage was observed in the regions analyzed by *en face* imaging.

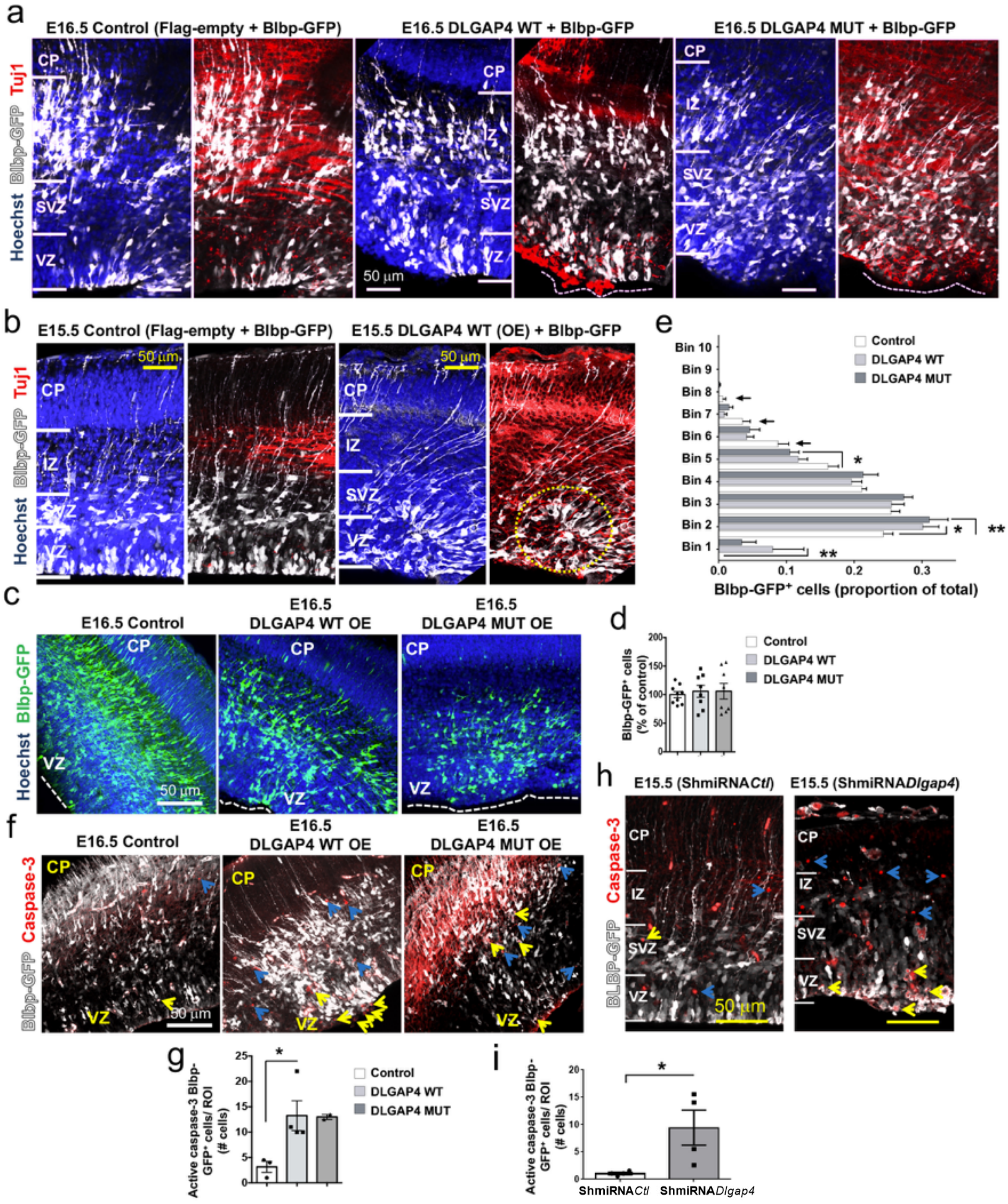

Supplementary Figure 7. Overexpression (OE) of WT and mutant (MUT) DLGAP4 reveals a ventricular surface phenotype. (**a**) Representative immunofluorescence showing Hoechst staining (blue), Blbp-GFP (white) and TuJ1^{+} (red) cells abnormally located at the ventricular surface phenotype in WT and MUT DLGAP4 OE brains (E16.5, electroporated at E14.5). (**b**) Rosette-like structures showing RG disruption in the WT DLGAP4 OE condition

at E15.5, indicated by a circle in a dotted line. (**c**) Representative images of Blbp-GFP cell distribution 48 h after IUE. (**d**) Blbp-GFP cell counts do not change significantly (n=8 embryos per condition). Quantification of data represents relativized individual values, mean ± SEM (one-way ANOVA with *post hoc* Tukey, $F_{2,21} = 0.036$, *n.s.* $p = 0.096$). (**e**) Blbp-GFP cell distribution. Two-way ANOVA with *post hoc* Sidak's: Interaction Bin x OE condition, $F_{18,210} = 2.28$, $p = 0.0029$; $^{*}p < 0.05$, $^{**}p = 0.0097$, $^{***}p = 0.0007$. Arrows show delayed migration trend from Bin 6 in both OE conditions. Bin 1 corresponds to the ventricular surface phenotype (only present in OE conditions). (**f**) Representative images of active caspase-3 (red) and Blbp-GFP (white) immunofluorescence at E16.5, electroporated at E14.5. Yellow arrows: double active caspase-3/Blbp-GFP^{+} cells; blue arrows: active caspase-3 cells. (**g**) Graph showing total active caspase-3 cells per ROI (n=3 *Ctl, n*=4 WT DLGAP4 and n=2 MUT DLGAP4 independent experiments per condition). Quantification data represent the individual values, mean ± SEM. Statistical analysis was performed using one-way ANOVA with *post hoc* Tukey, $F_{2,6} = 5.44$, $p = 0.045$; $^{*}p = 0.049$, ShmiRNA*Ctl* vs. WT DLGAP4 OE. (**h**) Representative images of active caspase-3 (red) and Blbp-GFP (white) immunofluorescence at E15.5 after KD experiments. Yellow arrows: double active caspase-3/Blbp-GFP^{+} cells; blue arrows: active caspase-3 cells. (**i**) Graph showing total active caspase-3 cells per ROI (n=4 independent experiments per condition). Quantification data represent the individual values, mean ± SEM. Statistical analysis was performed using two-sided unpaired t-test, $^{*}p = 0.039$, ShmiRNA*Ctl* vs. ShmiRNA*Dlgap*4.

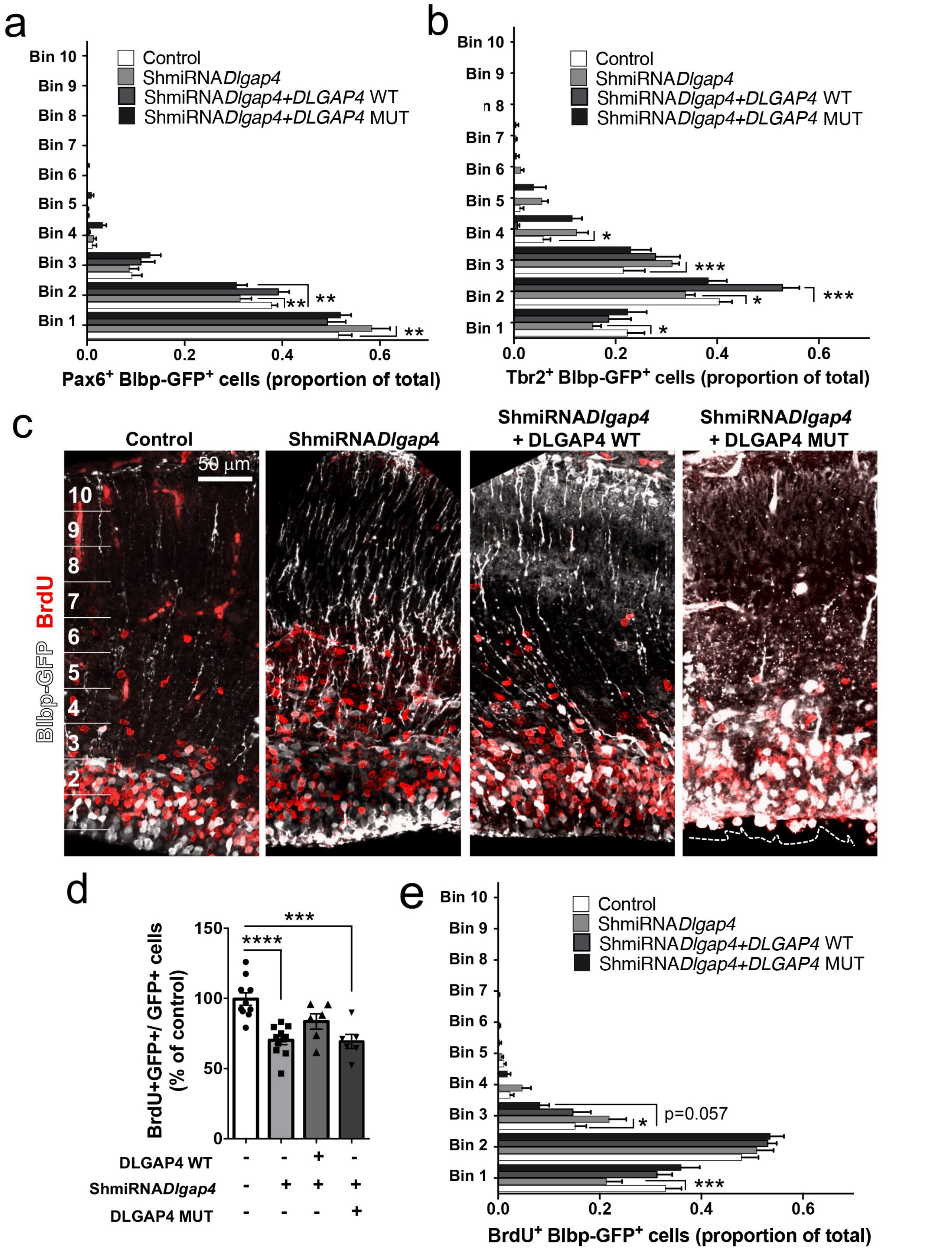

Supplementary Figure 8. WT but not MUT DLGAP4 restore cell proliferation defects after *Dlgap4* KD. **(a)** Pax6^{+}Blbp-GFP cells distribution. Quantification of data represents the mean ± SEM (n=10 *Ctl,* n=8 for ShmiRNA*Dlgap4*, ShmiRNA*Dlgap4*+DLGAP4 WT and

ShmiRNA*Dlgap4*+DLGAP4 MUT, embryos from at least 3 litters). Two-way ANOVA with *post hoc* Sidak's was performed: interaction Bin x expression factor $F_{27,300}$= 2.24, $p = 0.0006$; ** $p < 0.005$. **(b)** $Tbr2^{+}Blbp\text{-}GFP^{+}$ cells distribution. Quantification of data represents the mean ± SEM (n=14 *Ctl,* n=15 for ShmiRNA*Dlgap4*, n=5 ShmiRNA*Dlgap4*+DLGAP4 WT and n=7 ShmiRNA*Dlgap4*+DLGAP4 MUT, embryos from at least 3 litters). Two-way ANOVA with post-hoc Sidak's was performed: interaction Bin x expression factor, $F_{27, 370}$= 3.48, $p < 0.0001$; * $p < 0.05$, *** $p < 0.001$. (**c**) Representative images showing E15.5 brain immunostaining of BrdU after 30 min pulse (IUE E14.5). Slashed line shows the ventricular surface phenotype. (**d**) $BrdU^{+}Blbp\text{-}GFP^{+}$ cell counts are rescued with WT DLGAP4 construct but not with the mutant. Quantification data represent relativized individual values, mean ± SEM (n=10 *Ctl,* n=11 ShmiRNA*Dlgap4*, n=6 ShmiRNA*Dlgap4*+DLGAP4 WT and n=6 ShmiRNA*Dlgap4*+DLGAP4 MUT, embryos from at least 3 litters). One-way ANOVA with *post hoc* Tukey's was performed, $F_{3, 29}$= 11.73, $p < 0.0001$; *** $p = 0.0004$, **** $p < 0.0001$. (**e**) $BrdU^{+}$ $Blbp\text{-}GFP^{+}$ cell distribution along the cortical wall is rescued by both DLGAP4 constructs. Two-way ANOVA with *post hoc* Sidak's was performed: Interaction Bin x expression factor, $F_{27, 290}$= 2.99, $p = 0.0014$; * $p= 0.02$, *** $p = 0.0001$.

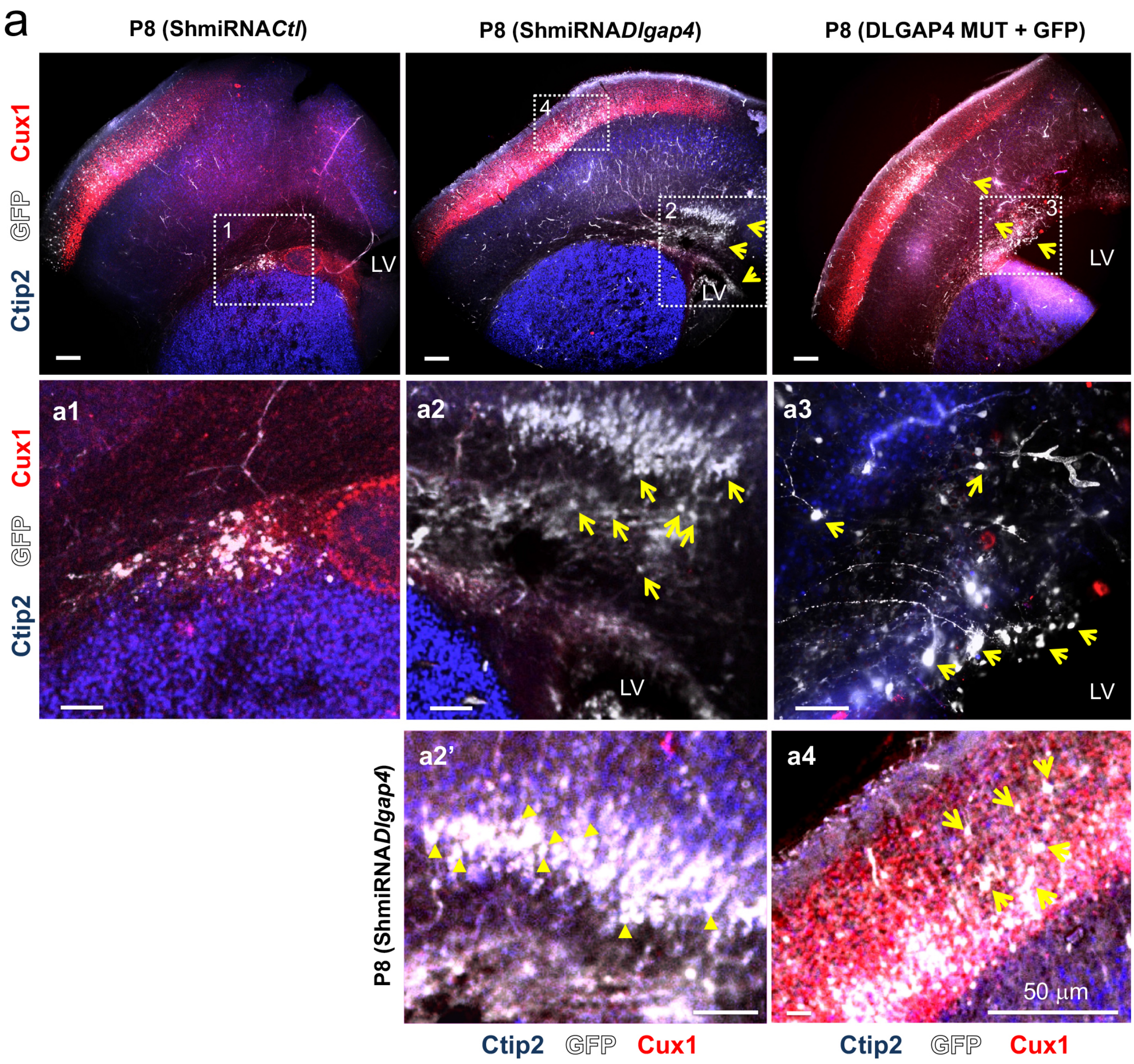

Supplementary Figure 9. ***Dlgap*4 KD leads to slowed migration and reduced cells reaching the CP by P8**. **(a)** Representative images showing Ctip2, Cux1 and GFP immunostainings in *Ctl, Dlgap4* KD and MUT DLGAP4 OE conditions at P8, co- electroporated with a Blbp-GFP vector at E14.5. Arrows indicate ectopic delayed migrating neurons (GFP^+ in Shmi*Dlgap*4) and ectopic cells at the VZ (GFP^+ in MUT DLGAP4 OE). (**a1-3**) Higher magnification of the dashed regions including the ventricular surface and the lateral ventricle (LV). (**a1**) In this control image, a small fraction of ectopic GFP^+ cells were also observed close to the ventricles. (**a2**) Co-immunostainings ($Cux1^+GFP^+$ or $Ctip2^+GFP^+$) showing the identity of ectopic GFP^+ cells in the Shmi*Dlgap*4 condition. Arrowheads indicate $Ctip2^+GFP^+$ cells. (**a4**) $Cux1^+GFP^+$ labeling

is reduced in some regions of the CP in Shmi*Dlgap4* brains due to reduced $Cux1^+$ cells. Arrows indicate $Cux1^-GFP^+$ cells.

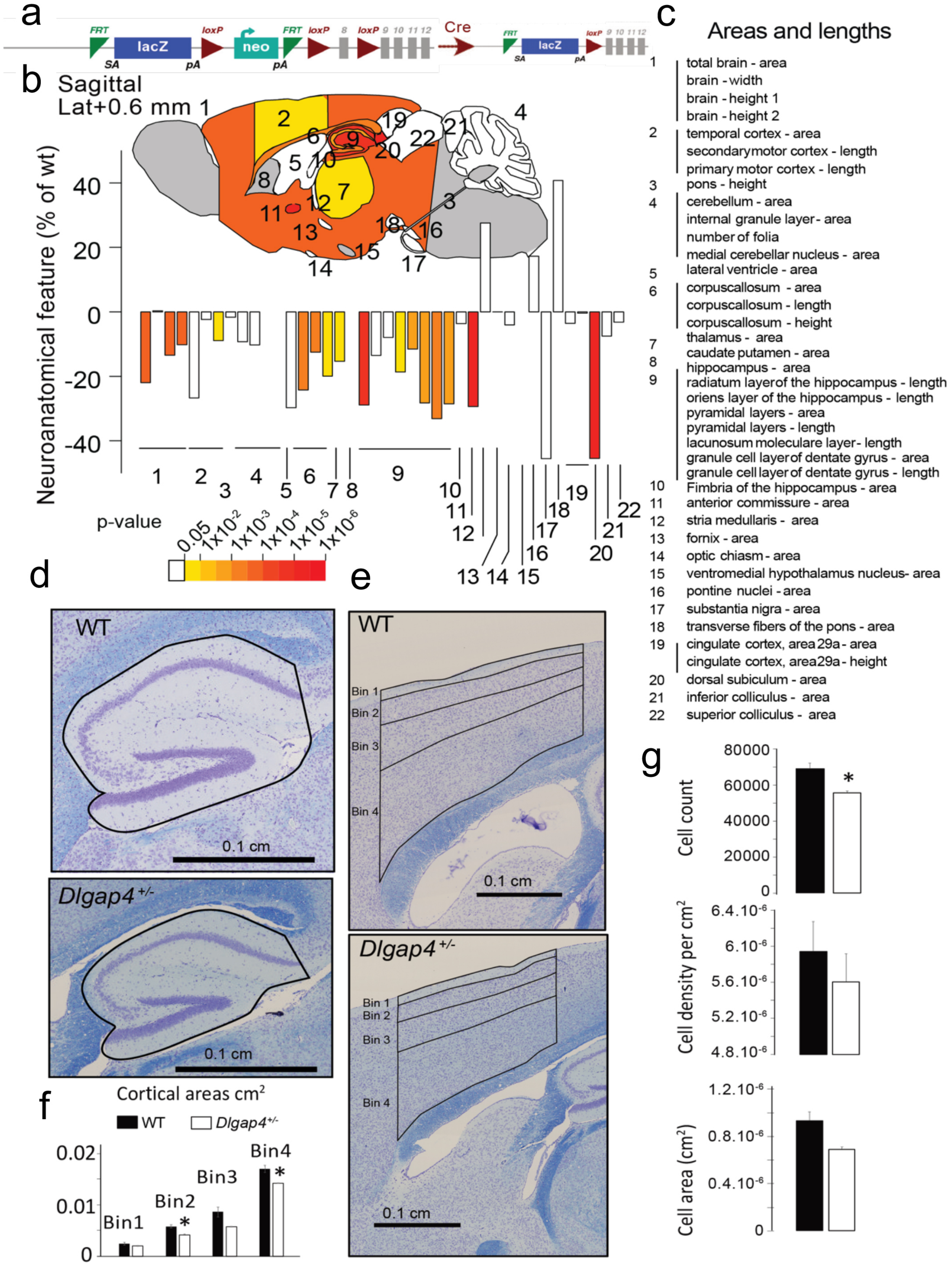

Supplementary Figure 10. Dlgap4 KO mouse studies show neuroanatomical defects in the dorsal telencephalon. (**a**) Construction of the *Dlgap4*$^{\mathrm{tm1a(KOMP)Wtsi}}$ and *Dlgap4*$^{\mathrm{tm1b(KOMP)Wtsi}}$

alleles, the latter being used in this study. (**b**) Top: Schematic representation of a section at lateral +0.60 mm. Colored regions indicate the presence of at least one significant parameter within the brain region at the 0.05 level. White coloring indicates a p-value higher than 0.05 and grey shows not enough data to calculate a p-value. Bottom: Histograms for 3 heterozygous *Dlgap4* mice showing variation (decreased-minus scale or increased-positive scale) in areas and lengths expressed as percentage of 3 WT together with a color map indicating the significance level. (**c**) List of area and length measurements assessed in the study. (**d**) Example of WT and heterozygous *Dlgap4* brain images in sagittal section double-stained for Nissl and Luxol showing the hippocampus. (**e**) Representative image of the cortex shown across the sagittal section at lateral +0.60 mm. (**f**) Quantification of four cortical areas in cm^2. Bin 1 corresponds to Layer 1, Bin 2 to layers II/IV, Bin 3 to layer V, and Bin 4 to layer VI. (**g**) Only male mice aged 16 weeks were used for neuroanatomical studies. Quantifications of cell count, cell density (cm^2) and average cell area (cm^2), * $p < 0.05$ Student's t-test of equal variance.
