# Supplementary material for: Novel role of the synaptic scaffold protein Dlgap4 in ventricular surface integrity and neuronal migration during cortical development
Source: Nat Commun. 2022 May 18;13:2746. doi: 10.1038/s41467-022-30443-z (PMC9117333; doi:10.1038/s41467-022-30443-z)
Supplement: Supplementary file 3 — Reporting Summary [file 41467_2022_30443_MOESM3_ESM.pdf]

nature portfolio

Corresponding author(s): Fiona Francis, NCOMMS-19-24706A

Last updated by author(s): 03/13/2022

Reporting Summary

Nature Portfolio wishes to improve the reproducibility of the work that we publish. This form provides structure for consistency and transparency in reporting. For further information on Nature Portfolio policies, see our Editorial Policies and the Editorial Policy Checklist.

Statistics

For all statistical analyses, confirm that the following items are present in the figure legend, table legend, main text, or Methods section.

n/a	Confirmed	
☐	☒	The exact sample size (n) for each experimental group/condition, given as a discrete number and unit of measurement
☐	☒	A statement on whether measurements were taken from distinct samples or whether the same sample was measured repeatedly
☐	☒	The statistical test(s) used AND whether they are one- or two-sided<br>*Only common tests should be described solely by name; describe more complex techniques in the Methods section.*
☒	☐	A description of all covariates tested
☐	☒	A description of any assumptions or corrections, such as tests of normality and adjustment for multiple comparisons
☐	☒	A full description of the statistical parameters including central tendency (e.g. means) or other basic estimates (e.g. regression coefficient) AND variation (e.g. standard deviation) or associated estimates of uncertainty (e.g. confidence intervals)
☐	☒	For null hypothesis testing, the test statistic (e.g. F, t, r) with confidence intervals, effect sizes, degrees of freedom and P value noted<br>*Give P values as exact values whenever suitable.*
☒	☐	For Bayesian analysis, information on the choice of priors and Markov chain Monte Carlo settings
☒	☐	For hierarchical and complex designs, identification of the appropriate level for tests and full reporting of outcomes
☒	☐	Estimates of effect sizes (e.g. Cohen's d, Pearson's r), indicating how they were calculated

Our web collection on statistics for biologists contains articles on many of the points above.

Software and code

Policy information about availability of computer code

Data collection	No software was used to collect data
Data analysis	Image J NIH, Prism Graph Pad software, Polyweb, University Paris Descartes, Imagine Institute. PolyPhen-2, PolyPhen-2. http://genetics.bwh.harvard.edu/pph2/ S). SIFT, Sorting Intolerant From Tolerant https://sift.bii.a-star.edu.sg/ S. Genome Aggregation Database (gnomAD), https://gnomad.broadinstitute.org/. I-TASSER, Iterative Threading ASSEmbly Refinement, ZhangLab, https://zhanggroup.org/I-TASSER/. Pymol, https://pymol.org/2/. siRNA Wizard™ Software, https://www.invivogen.com/sirna-wizardtm-software. gnomAD, Genome Aggregation Database, https://gnomad.broadinstitute.org/. Single-cell transcriptomic atlas of the developing neocortex, http://genebrowser.unige.ch/science2016/. CoDEx Viewer, (Cortical Development Expression Viewer), http://solo.bmap.ucla.edu/shiny/webapp/#, CellProfiler, cell image analysis software, https://cellprofiler.org/.

For manuscripts utilizing custom algorithms or software that are central to the research but not yet described in published literature, software must be made available to editors and reviewers. We strongly encourage code deposition in a community repository (e.g. GitHub). See the Nature Portfolio guidelines for submitting code & software for further information.

Data

Policy information about availability of data

All manuscripts must include a data availability statement. This statement should provide the following information, where applicable:

- Accession codes, unique identifiers, or web links for publicly available datasets
- A description of any restrictions on data availability
- For clinical datasets or third party data, please ensure that the statement adheres to our policy

All data are available from the authors upon reasonable request

Field-specific reporting

Please select the one below that is the best fit for your research. If you are not sure, read the appropriate sections before making your selection.

- [x] Life sciences
- [ ] Behavioural & social sciences
- [ ] Ecological, evolutionary & environmental sciences

For a reference copy of the document with all sections, see nature.com/documents/nr-reporting-summary-flat.pdf

Life sciences study design

All studies must disclose on these points even when the disclosure is negative.

Sample size	The sample size selection for experiments was based on both published and previous pilot studies considering the sensitivity of the applied approaches.
Data exclusions	In IUE experiments, brain samples with less of 75 electroporated cells were excluded from the data analysis as indicated in the Methods section.
Replication	Independent experiments were carried out to ensure reproducibility of experimental outcomes.
Randomization	Pregnant mice/embryos were randomly assigned to the different DNA constructs as mentioned in the Statistical Analysis section of the Methods.
Blinding	When possible, data were collected and analyzed in a blind manner to the experimenter as stated in the Statistical Analysis section of the Methods.

Reporting for specific materials, systems and methods

We require information from authors about some types of materials, experimental systems and methods used in many studies. Here, indicate whether each material, system or method listed is relevant to your study. If you are not sure if a list item applies to your research, read the appropriate section before selecting a response.

Materials & experimental systems

n/a	Involved in the study	
[ ]	[x]	Antibodies
[ ]	[x]	Eukaryotic cell lines
[x]	[ ]	Palaeontology and archaeology
[ ]	[x]	Animals and other organisms
[x]	[ ]	Human research participants
[ ]	[x]	Clinical data
[x]	[ ]	Dual use research of concern

Methods

n/a	Involved in the study	
[x]	[ ]	ChIP-seq
[x]	[ ]	Flow cytometry
[ ]	[x]	MRI-based neuroimaging

Antibodies

Antibodies used

Rabbit anti-DLGAP4 (Cat#ab73285, Abcam, RRID: AB_1268552) 1:500 (WB, ICC)
Mouse anti–Ki-67 (Cat#556003, BD Bioscience, RRID: AB_396287) 1:200 (IHC)
Mouse anti–β-catenin (Cat#c19220, BD Bioscience, RRID: AB_2341177) 1:500 (IHC)
Mouse anti-N-cadherin (Cat#610920, clone 32, BD Biosciences, RRID: AB_398236) 1:500 (IHC)
Mouse anti-Nestin (Clone 2Q178, cat# ab6142, Abcam, RRID: AB_305313) 1:600 (IHC)
Rabbit anti-FLNA (Cat# A301-135A, Bethyl, RRID: AB_2106430) 1:2000 (WB)
Rabbit anti-p38 (Cat# SPC-172, StressMarq Bioscience, RRID: AB_1055444) 1:1000 (WB)
Rabbit anti-Raptor (Cat# ab5454, Abcam, RRID: AB_304901) 1:2000 (WB)
Rabbit anti-Rictor (A300-459A, Bethyl, RRID: AB_2179967) 1:2000 (WB)
Mouse anti-DLG1 (clone S64-15, Cat#MAB8585, Abnova, RRID: AB_10719194) 1:1000 (WB); 1:500 (ICC, IHC)
Mouse anti-Cux1 (M-222, Cat#sc13024, Santa Cruz Biotechnology) 1:1000 (IHC)
Mouse anti-Class III β3 tubulin (mouse clone TUJ1, Cat#MMS-435P, Covance) 1:10000 (IHC)
Rat anti-BrdU (clone BU1/75 (ICR1), Cat# MCA2060T, Bio-Rad, RRID: AB_10015293) 1:1000 (IHC)
Rat anti- Ctip2 (clone 25B6, Cat#ab18465, Abcam, RRID: AB_2064130) 1:500 (IHC)
Rabbit anti–caspase-3 (Cat# 559565, BD Biosciences, RRID: AB_397274) 1:250 (IHC)
Rabbit anti-Tbr2 (Cat# ab23345, Abcam, RRID: AB_778267) 1:300 (IHC)
Rabbit anti-PH3 (Ser10) (Cat# 06-570, Millipore, RRID: AB_310177) 1:400 (IHC)
Chicken anti-GFP (Cat# AB16901, Millipore, RRID: 90890) 1:500 (IHC)
Chicken anti-Tbr2 (Cat# AB15894, Millipore, RRID: AB_10615604) 1:200 (IHC)

Mouse anti-GFP (Clone GFP-20, Cat#G6539, Sigma, RRID: AB_259941) 1:1000 (WB); 1:500 (ICC, IHC)
Rabbit anti-GFP (Cat#A6455, Invitrogen RRID: AB_221570) 1:500 (IHC)
Rabbit anti-Flag (Cat#F7425, Sigma, RRID: AB_439687) 1:300 (WB)
Mouse anti-Flag (Clone M2, Cat#F1804, Sigma, RRID: 262044) 1:500 (WB)
Mouse anti-c-myc (Clone 9E10, Cat#9B11, Cell Signaling Technology, RRID: AB_10889248) 1:400 (WB)
Chicken anti-GAPDH (Cat#AB2302, Millipore, RRID: AB_10615768) 1:2000 (WB)
Rat anti-HA (clone 3F10, Cat#11815016001, Roche, RRID: AB_390914) 1:300 (WB)
Rabbit anti-Pax6 (Cat#901301, BioLegend, RRID: AB2565003) 1:300 (IHC)
Goat anti-mouse Alexa 488 (Cat#A28175, Thermo Fisher Scientific, RRID: AB_2536161) 1:1000 (ICC)
Goat anti-rabbit Alexa 568 (Cat#A-11011, Thermo Fisher Scientific, RRID: AB_143157) 1:1000 (ICC)
Goat anti-mouse Alexa 568 (Cat#A-11031, Thermo Fisher Scientific, RRID: AB_144696) 1:1000 (ICC)
Goat anti-rabbit Alexa 488 (Cat#A-11008, Thermo Fisher Scientific, RRID: AB_143165) 1:1000 (ICC)
Goat anti-mouse Alexa 555 (Cat#A32727, Thermo Fisher Scientific, RRID: AB_2633276) 1:1000 (ICC)
Goat anti-rat Alexa 568 (Cat#A-11006, Thermo Fisher Scientific, RRID: AB_141373) 1:1000 (ICC)
Donkey anti-rabbit Alexa 647 (Cat#A-31573, Thermo Fisher Scientific, RRID: AB_2536183) 1:1000 (ICC)
Goat anti-chicken Alexa 568 (Cat# A-11041, Thermo Fisher Scientific; RRID:AB_2534098) 1:1000 (ICC)
DyLight anti-mouse 800 (Cat#SA5-35521, Thermo Fisher Scientific, RRID: AB_2556774) 1:10000 (WB)
DyLight anti-rabbit 800 (Cat#SA5-35571, Thermo Fisher Scientific, RRID: AB_2556775) 1:10000 (WB)
DyLight anti-chicken 680 (Cat#SA5-10074, Thermo Fisher Scientific, RRID: AB_2556654) 1:10000 (WB)
DyLight anti-mouse 680 (Cat#039610144121, Rockland, RRID: AB_1057546) 1:10000 (WB)
DyLight anti-rabbit 680 (Cat#611-144-002, Rockland, RRID: AB_1660962) 1:10000 (WB)
DyLight anti-rat 680 (Cat# SA5-10022, Thermo Fisher Scientific, RRID: AB_2556602) 1:10000 (WB)

Validation

All antibodies used in our study were previously validated by their respective manufacturers. Only, in the case of the DLGAP4 (Ab73285, Abcam) antibody, we performed a thorough validation for IHC using peptide competition assays as detailed in the Methods section. Briefly, two consecutive 70 µm brain slices from E14.5 embryos were used for each experiment. Rabbit anti-DLGAP4 antibody (1µg/ml, Ab73285, Abcam) was diluted at a concentration of 1:500 in blocking solution (PBS, 10% NGS, 0.1% triton X-100). The solution was then divided into two tubes. In the first tube, labeled "blocked", respectively 5X or 100X excess of DLGAP4 blocking peptide (0.2mg/ml, Ab219578, Abcam) were added. In the second tube, labeled "control", an equivalent amount of buffer was added. Both tubes were then incubated, with agitation, overnight at 4°C. The immunofluorescence procedure was performed as described in the manuscript. The labeling that disappears when using the "blocked" antibody corresponds to the specific labeling of the DLGAP4 primary antibody. The images were acquired using an Olympus FV10i confocal microscope (shown in Suppl. Figure 2).

Eukaryotic cell lines

Policy information about cell lines

Cell line source(s)

Human: hTERT RPE1 ATCC (CRL-4000); Mouse: Neuro2A ATCC (CCL-131)

Authentication

None of the cell lines used were authenticated further

Mycoplasma contamination

Mycoplasm contamination was tested rigorously every month by PCR from samples of cultured cell media. We did not record any Mycoplasm contaminations during our experiments.

Commonly misidentified lines
(See ICLAC register)

N/A

Animals and other organisms

Policy information about studies involving animals; ARRIVE guidelines recommended for reporting animal research

Laboratory animals

Mouse: SWR/J pregnant female mice of 8 weeks (Janvier Labs, France); Dlgap4tm1b(KOMP)Wtsi male and female mice of 16 weeks (Wellcome Sanger Institute, UK).

Wild animals

N/A

Field-collected samples

N/A

Ethics oversight

Charles Darwin Committee Paris (French MESR 00984.02), France; Wellcome Sanger Institute Animal Welfare and Ethical Review Body (Act of 1986 under UK Home Office Lic. 80/2076), UK.

Note that full information on the approval of the study protocol must also be provided in the manuscript.

Clinical data

Policy information about clinical studies

All manuscripts should comply with the ICMJE guidelines for publication of clinical research and a completed CONSORT checklist must be included with all submissions.

Clinical trial registration	N/A
Study protocol	Following standard protocols, patient DNA or blood samples and informed consent (from the patients' parents) were obtained according to the guidelines of local institutional review boards (IRBs APHP-Délégation Interrégionale à la Recherche Clinique, from different French hospitals). Informed consent was obtained from each patient included in this study and the study protocol conformed to the ethical guidelines of the 1975 Declaration of Helsinki as reflected in a priori approval by the institution's human research committee (Comité de Protection des Personnes, different French hospitals). Both males and females were included in this study, for diagnostic purposes, and there was no participant compensation.
Data collection	N/A
Outcomes	N/A

Magnetic resonance imaging

Experimental design

Design type	MRI were performed for diagnosis purposes
Design specifications	*Specify the number of blocks, trials or experimental units per session and/or subject, and specify the length of each trial or block (if trials are blocked) and interval between trials.*
Behavioral performance measures	N/A

Acquisition

Imaging type(s)	Structural
Field strength	1.5 Tesla
Sequence & imaging parameters	Volume T1 gradient echo sequence, 3D-T2 fluid-attenuated inversion recovery (FLAIR) and 3D- Double inversion recovery (DIR) with reconstruction in the three planes. 3D-T2 coronal sequence.
Area of acquisition	3 sections (axial, coronal and sagittal sections) allowed a good evaluation of the temporal lobe.

Diffusion MRI ☐ Used ☒ Not used

Preprocessing

Preprocessing software	N/A
Normalization	N/A
Normalization template	N/A
Noise and artifact removal	N/A
Volume censoring	N/A

Statistical modeling & inference

Model type and settings	N/A
Effect(s) tested	N/A

Specify type of analysis: ☒ Whole brain ☐ ROI-based ☐ Both

Statistic type for inference (See Eklund et al. 2016)	N/A
Correction	N/A

Models & analysis

n/a	Involved in the study
☒	☐ Functional and/or effective connectivity
☒	☐ Graph analysis
☒	☐ Multivariate modeling or predictive analysis
